# Swallowing Disorders in Ischemic Stroke: A Retrospective Analysis of Dysphagia Severity Differences Based on Hemisphere Damage and Patient Age

**DOI:** 10.3390/jcm14030900

**Published:** 2025-01-29

**Authors:** Anna Żdanowicz, Hanna Sobkowiak, Maciej Gawrysiak, Małgorzata Wiszniewska

**Affiliations:** 1 Department of Nursing, University of Applied Sciences, 64-920 Piła, Poland; azdanowicz@ans.pila.pl; 2 Neurological Department with Stroke Treatment Subdivision, Specialist Hospital, 64-920 Piła, Poland; hania_urbanowska@wp.pl (H.S.); mpwisz@gmail.com (M.W.); 3 Department of Economics, University of Applied Sciences, 64-920 Piła, Poland; 4 Department of Emergency Medical Services, University of Applied Sciences, 64-920 Piła, Poland

**Keywords:** ischemic stroke, dysphagia, swallowing disorders

## Abstract

**Background/Objectives:** Management of the acute phase of ischemic stroke requires intensive interdisciplinary collaboration among medical personnel. Dysphagia, or swallowing disorders, represents a serious complication of stroke. Accurate diagnosis and identification of the causes of dysphagia are essential for implementing effective treatment. The aim of the study was to investigate the impact of age, acute stroke treatment, localization, comorbidities, and early rehabilitation on the severity and progression of dysphagia in patients with acute ischemic stroke. **Methods:** The study analyzed the severity and progression of dysphagia in patients with ischemic stroke, considering patient age, stroke localization, comorbidities, and the impact of thrombolytic therapy and early rehabilitation on swallowing outcomes. **Results:** Severe dysphagia was more common among older patients and those with cardiovascular conditions. Early rehabilitation was particularly effective in improving swallowing in right hemisphere strokes, while thrombolytic therapy demonstrated benefits for patients under 65 years. **Conclusions:** Therapy led to an improvement in the condition of all patients with dysphagia following ischemic stroke. Further prospective research is needed to better understand swallowing disorders and optimize treatment.

## 1. Introduction

A stroke is a clinical syndrome characterized by the sudden onset of generalized or localized symptoms of vascular origin, resulting in brain function disorders. These symptoms typically persist for more than 24 h unless the patient dies earlier. However, stroke may also include cases where characteristic clinical symptoms last less than 24 h, provided that neuroimaging reveals an ischemic lesion corresponding to these symptoms [1,2,3,4].

One of the complications of ischemic stroke (IS) is dysphagia, a condition that causes difficulties in swallowing, including the intake and movement of food from the mouth to the pharynx, esophagus, and stomach. A rapid assessment of swallowing, performed by a physician, nurse, or speech therapist, is critical to minimizing the risk of complications associated with dysphagia, particularly aspiration pneumonia, which is one of the most severe complications of IS [5,6,7].

In individuals with swallowing disorders, symptoms related to food intake and the consequences of inadequate nutrition are frequently observed.

Aspiration pneumonia is one of the most serious complications associated with swallowing disorders. Prompt treatment is essential to minimize the risk of respiratory failure or pulmonary edema [5,6,8,9].

The evaluation of swallowing function places significant emphasis on the assessment of cranial nerves, as they play a critical role in the swallowing process [10,11,12,13].

Swallowing disorders are frequently observed during the acute phase of stroke. Therefore, it is recommended that all patients undergo dysphagia screening within 24 h of hospital admission. Patients who test positive should be referred for a comprehensive swallowing evaluation by a speech therapist [6].

Screening tests are essential for identifying individuals who require speech therapy and for implementing appropriate therapeutic and nutritional interventions. These measures aim to improve swallowing function and reduce the risk of aspiration pneumonia, malnutrition, and dehydration [12,14]. Examples of screening tests for dysphagia include the 90 mL water test and the Gugging Swallowing Screen (GUSS) [4,12,14,15,16].

The prognosis and rehabilitation of patients with dysphagia following a stroke depend on several factors, including the time of symptom onset, the time of hospital admission, the accuracy of diagnosis, and the speed of initiating treatment [6,9,11,16]. In dysphagia therapy, controlling body and head posture, as well as assessing sensory, motor, and respiratory function, plays a key role. The therapeutic methods employed focus on enabling oral intake of food while protecting the airways from aspiration [17,18].

The aim of the study was to investigate the impact of age, acute stroke treatment, localization, comorbidities, and early rehabilitation on the severity and progression of dysphagia in patients with acute ischemic stroke.

## 2. Methods

### 2.1. Organization of Scientific Research

The study used a retrospective analysis of the medical records of patients treated in the Neurology Department with the Stroke Treatment Subdivision at the Stanisław Staszic Specialist Hospital in Piła between 1 November 2023 and 30 April 2024.

Eighty patients were included in the analysis, all of whom were diagnosed with stroke within the first 24 h of symptom onset based on clinical examination and computed tomography (CT) and underwent a swallowing test within the first 24 h after admission. Patients received either thrombolytic treatment or “classical” treatment, depending on their qualification.

### 2.2. Research Procedures

The assessment of swallowing status was conducted within the first 24 h after the onset of symptoms and on the seventh day of hospitalization. Changes in swallowing function were analyzed, with particular attention to differences related to the location of brain damage. Nursing observation involved collecting information about the patient’s behavior and evaluating their condition during the screening test for swallowing disorders.

The analysis of medical records included gathering data related to diagnosis, diagnostic test results (such as CT and Magnetic Resonance Imaging—MRI), medical and nursing history, discharge summary, vital signs, treatment applied, and medical recommendations.

The assessment of swallowing disorders was conducted using the standardized GUSS test, which consists of an initial part and three stages for evaluating the swallowing of foods with different consistencies. Points are awarded based on the difficulty in swallowing:

- 0–9 points: Severe dysphagia, significant difficulty in swallowing, high risk of aspiration;
- 10–14 points: Moderate dysphagia, moderate risk of aspiration;
- 15–19 points: Mild dysphagia, low risk of aspiration;
- 20 points: No swallowing disorders, minimal risk of aspiration.

Before the study began, approval was obtained from the hospital director for the analysis of medical records. All procedures complied with the ethical principles applicable to scientific research.

### 2.3. Characteristics of the Study Population

A total of 80 patients were included in the study, with 50% (*n* = 40) being patients with left hemisphere stroke, and the remaining 50% (*n* = 40) with right hemisphere stroke. Women participating in the study accounted for 51.2% (*n* = 41) of the total, while men made up 48.8% (*n* = 39). The patients with right and left hemisphere strokes were similar in age. The average age of patients with left hemisphere stroke was 72.1 (±14.3) years, with a median of 70 years (the youngest patient was 29 years old, and the oldest was 99 years old). In the group of patients with right hemisphere stroke, the average age was 71.6 (±11.7) years, with a median of 73 years. The youngest person was 40 years old, and the oldest was 100. The largest group (40%; *n* = 32) consisted of individuals aged 67 to 76 years (Table 1).

### 2.4. Statistical Analysis

Statistical analyses were performed using R (v. 4.4.2), PQStat (v. 1.8.6), and Excel (v. 2016). Quantitative data were summarized with descriptive statistics, while qualitative data were presented as frequencies and percentages. Tests included Shapiro–Wilk for normality, Mann–Whitney U for two-group comparisons, Kruskal–Wallis ANOVA for multiple groups, and chi-squared tests for associations. Spearman’s correlation coefficients and logistic regression were used to analyze relationships, with significance set at *p* < 0.05.

## 3. Results

On the first day, the average GUSS score was 13.37 (±6.71) for left hemisphere stroke patients and 14.27 (±7.02) for right hemisphere stroke patients, with no statistically significant difference. Moderate-to-severe dysphagia was observed in 52.5% of left hemisphere stroke patients and 40% of right hemisphere stroke patients (Table 2).

On the seventh day, the average GUSS scores were higher compared to the first day. On day 7, the average score for patients with left hemisphere stroke was 16.02 (±6.41), with a median of 19 points (minimum score—0 points, maximum—20 points). For patients with right hemisphere stroke, the average score was 17.25 (±4.90), with a median of 20 points (minimum score—3 points, maximum—20 points). Moderate-to-severe dysphagia on the seventh day was present in 27.5% (*n* = 11) of patients with left hemisphere stroke and in 22.5% (*n* = 9) of those with right hemisphere stroke (Table 3).

Compared to the first day, the number of patients with resolving dysphagia, as well as those in whom dysphagia had completely resolved, increased by the seventh day. While significant improvements in swallowing function are observed in patients with right hemisphere stroke, it is crucial to compare the proportion of patients who achieved full recovery of swallowing ability, as measured by a GUSS score of 20. In this regard, nine patients with right hemisphere stroke fully regained their swallowing capability, compared to only seven patients with left hemisphere stroke. This highlights the importance of introducing a measure to quantify the difference in GUSS scores between the first and seventh day, which will be detailed in subsequent sections of this study.

### 3.1. Dependence Analysis Between Patient Age and Dysphagia Severity on Days 1 and 7 Post Stroke

In the group of patients with left hemisphere stroke (LHS) on Day 7, moderate-to-severe dysphagia was diagnosed in 53.3% (*n* = 8) of patients aged 75 years and older, whereas absent-to-mild dysphagia was observed in the majority (88%; *n* = 22) of patients aged 74 years and younger. Among patients with right hemisphere stroke (RHS), moderate-to-severe dysphagia affected 13.3% (*n* = 2) of individuals aged 75 years and older, while absent-to-mild dysphagia was noted in 72% (*n* = 18) of patients in the younger age group (under 75 years). The age-dependent relationship with dysphagia for left hemisphere stroke was statistically significant, as determined by the chi-squared test (*p* = 0.01) (Table 4).

The analysis of the relationship between dysphagia severity on the first day and patient age revealed a negative correlation (r = −0.35, *p* < 0.001). The findings indicate that older patients tend to experience more severe swallowing disorders. Similar results were observed on the seventh day (r = −0.37, *p* < 0.001).

The analysis demonstrated a significant effect of GUSS scores on Day 7 and the improvement in GUSS scores (Day 1 to Day 7) for patients with mild or absent dysphagia. GUSS scores on Day 7 strongly influenced the odds of mild dysphagia (OR = 0.115, 95% CI: 0.0107–1.24, *p* = 0.07), and the improvement in GUSS scores was statistically significant (OR = 0.432, 95% CI: 0.199–0.936, *p* = 0.03). However, no significant effects were observed for moderate/severe dysphagia, as neither GUSS scores on Day 7 (OR = 0.921, *p* = 0.12) nor the improvement in GUSS scores (OR = 1.12, *p* = 0.2) reached statistical significance (Table 5).

These findings suggest that age plays a crucial role in the progression and resolution of mild dysphagia. Further studies may focus on tailored interventions for older stroke patients to improve their swallowing outcomes.

### 3.2. Correlation Analysis Between Stroke Severity According to the NIHSS (National Institutes of Health Stroke Scale) and Dysphagia Severity on Days 1 and 7 Post Stroke

The average NIHSS stroke severity score was 7.85 (±4.76) for left hemisphere strokes and 10.12 (±6.75) for right hemisphere strokes, with moderate or severe dysphagia prevalence increasing alongside stroke severity. Severe dysphagia was observed in 100% of patients with moderate-to-severe left hemisphere strokes and up to 75% of patients with severe right hemisphere strokes (Table 6).

The analysis showed a significant negative correlation between stroke severity (NIHSS—numerical) and dysphagia severity (GUSS—numerical) on both Day 1 (r = −0.53, *p* < 0.001) and Day 7 (r = −0.44, *p* < 0.001), indicating that more severe strokes were associated with greater swallowing disorders.

A logistic regression analysis showed a significant association between NIHSS stroke severity and moderate/severe dysphagia on Day 1 (*p* = 0.03), with a 2.11-fold increase in odds for each one-level rise in NIHSS. This association was stronger on Day 7 (*p* = 0.016), where the odds increased by 2.44 times (95% CI: 1.18–5.05), as shown in Table 7.

### 3.3. Analysis Between Speech Disorders and Hemispere Stroke

In the group of patients diagnosed with right hemisphere stroke, 60% (*n* = 24) had no speech disorders, while in the group with left hemisphere stroke, 30% (*n* = 12) were without speech disorders.

A logistic regression analysis was conducted to evaluate the association between stroke hemisphere (0 for right hemisphere, 1 for left hemisphere) and the presence of aphasia or dysarthria (0 for no aphasia or dysarthria, 1 for aphasia or dysarthria). The results are shown below in Table 8.

Stroke hemisphere (1 for left hemisphere, 0 for right hemisphere) was a statistically significant predictor. Left hemisphere stroke significantly increased the odds of aphasia or dysarthria by 3.5 times (OR = 3.5, 95% CI: 1.39–8.84, *p* = 0.008).

### 3.4. Analysis Between the Use of Thrombolysis and Dysphagia Severity on Days 1 and 7 Post Stroke

Thrombolytic treatment was administered to 47.5% (*n* = 19) of patients with left hemisphere stroke and to 20% (*n* = 8) of patients with right hemisphere stroke. In the group of individuals with left hemisphere stroke, 26.3% (*n* = 5) of those who received thrombolysis had severe dysphagia on the first day, compared to 33.3% (*n* = 7) of patients without thrombolytic treatment.

The impact of thrombolytic treatment on the course of dysphasia is shown in Table 9. These results suggest that thrombolytic treatment may have positively influenced the recovery of patients under 65 years of age.

### 3.5. Association Between Comorbidities and Dysphagia Severity

The relationship between comorbidities and dysphagia severity is crucial for understanding the recovery trajectory in stroke patients. Comorbid conditions may influence both the baseline swallowing function (measured on Day 7 with GUSS) and the improvement in swallowing over time (measured as the difference between GUSS scores from Day 1 to Day 7). This analysis examines the association between specific comorbidities and dysphagia severity (Table 10).

The analysis identified significant associations between certain comorbidities and dysphagia severity. A history of previous stroke and atherosclerosis was linked to reduced swallowing function improvement and worse outcomes on Day 7, while ischemic heart disease (*p* = 0.006) and atrial fibrillation (*p* = 0.004) were also associated with greater dysphagia severity. Conditions like diabetes, hypertension, and hypothyroidism showed no significant impact. These findings highlight the need for careful management of comorbidities affecting swallowing in stroke patients.

## 4. Discussion

Dysphagia is a serious complication in patients after stroke, as confirmed by analyses conducted in various countries [5,6,7,9,13,18,19]. A Brazilian study, based on a retrospective analysis of medical records from 596 patients, found that dysphagia occurred in 19.6% of patients with ischemic stroke, of which 91.5% of cases were mild and 8.5% were severe. A correlation was noted between dysphagia and older age, hypertension, diabetes, and brainstem injuries. In most patients, dysphagia had a mild course, and most regained the ability to swallow normally within approximately 2.4 months [9].

The analysis of our own results confirms that dysphagia was similarly frequent in both left and right hemisphere strokes, with a higher frequency of severe type among older patients aged 75 years and over. In the conducted study, 52.5% of patients with left hemisphere stroke and 40% with right hemisphere stroke experienced moderate-to-severe dysphagia, but this difference was not statistically significant. This percentage was slightly higher than in other studies, where it was approximately 30% [7,9,13,16,19,20,21]. Improvement in dysphagia was noticeable on the seventh day post stroke, with a greater frequency of resolution in patients with right hemisphere stroke, but this difference was not statistically significant. A study in Iran, which examined 137 patients, found that dysphagia occurred in 31.4% of patients with acute ischemic stroke and was more common in cases of left hemisphere stroke. This study also emphasizes the importance of age as a risk factor for dysphagia [19].

Studies conducted in the United States at the University of Wisconsin–Madison Hospital showed that dysphagia occurred in 32% of patients, while dysarthria and aphasia affected 26% and 16% of patients, respectively. The high prevalence of dysphagia and verbal communication disorders underscores the importance of early specialized rehabilitation to minimize these disorders as quickly as possible [13]. All our patients also underwent early specialized rehabilitation, and on the seventh day, fewer patients had severe dysphagia, which included 27.5% of patients with left hemisphere stroke and 22.5% with right hemisphere stroke. A study conducted in Vietnam highlighted the benefits of daily swallowing exercises, which improved dysphagia in patients fed through a nasogastric tube. The results indicated a 20% advantage in the effectiveness of the exercises compared to routine care [18]. The results of our study are similar to the data in the literature, confirming the impact of stroke severity and patient age on dysphagia severity, as well as the relationship between speech disorders and dysphagia.

It is worth noting that, despite rehabilitation, in the group of older patients, on the seventh day, more patients had severe dysphagia compared to patients under 75 years of age, and this difference was statistically significant.

Among our patients, cardiovascular diseases showed a significant impact on the severity of dysphagia, and this relationship was statistically significant.

The findings of our study align with the results presented in [22], which demonstrated that thrombolytic therapy can lead to significant improvements in oral dietary intake and shorter hospital stays in post-ischemic stroke patients. Similarly to their results, we observed a positive impact of thrombolytic therapy on dysphagia improvement, particularly in patients younger than 65 years. While our study highlights this benefit in the context of early rehabilitation, the referenced research further underscores the value of thrombolytic therapy in improving functional outcomes related to swallowing and reducing hospitalization duration. These complementary findings emphasize the need for further research on the mechanisms by which thrombolytic therapy may influence dysphagia outcomes across different patient populations

### Study Limitations and Suggestions for Future Research

Our study has certain limitations: a short observation period and a relatively small number of patients. A larger sample size could more accurately reveal the relationships between comorbidities and the occurrence of dysphagia. An interesting direction for future research could also be investigating the impact of oral exercises on reducing dysphagia. Furthermore, it seems advisable to study a larger number of patients treated with thrombolysis in terms of dysphagia occurrence. There is a need for more prospective research to fully understand the mechanisms influencing swallowing disorders in order to organize and implement the most effective therapy and rehabilitation.

## 5. Conclusions

In ischemic stroke, severe dysphagia was found to be more prevalent among older patients and those with coexisting cardiovascular conditions, such as previous stroke, ischemic heart disease, and atrial fibrillation. Early dysphagia rehabilitation proved to be more effective in improving swallowing function in patients with right hemisphere stroke, highlighting the importance of targeted therapeutic interventions. Additionally, thrombolytic treatment showed a beneficial effect on the early improvement of dysphagia in patients younger than 65 years, suggesting the potential value of timely medical interventions in this population. There is a need for more prospective research to fully understand the mechanisms influencing swallowing disorders.

## Figures and Tables

**Table 1:** Demographic data.

Variable	*n*	%
Gender		
Woman	41	51.2
Man	39	48.8
Age groups		
<75 years	50	62.5
75 and more	30	37.5
Type of stroke		
Left hemisphere	40	50
Right hemisphere	40	50

Source: Own research results

**Table 2:** Comparative characteristics of GUSS values on the first day of stroke depending on the damaged hemisphere.

Variable	Left Hemisphere Stroke		Right Hemisphere Stroke	
	*n*	%	*n*	%
GUSS on Day 1				
0–14 Moderate-to-severe dysphagia	21	52.5	16	40
15–20 Absent-to-mild dysphagia	19	47.5	24	60
chi-squared *p*-value	0.37			

**Table 3:** Comparative characteristics of GUSS values on the seventh day of stroke depending on the damaged hemisphere.

Variable	Left Hemisphere Stroke		Right Hemisphere Stroke	
	*n*	%	*n*	%
GUSS on Day 7				
0–14 Moderate-to-severe dysphagia	11	27.5	9	22.5
15–20 Absent-to-mild dysphagia	29	72.5	31	77.5
chi-squared *p*-value	0.796			

**Table 4:** Comparative characteristics of dysphagia, divided by age groups.

Variable	Dysphagia on Day 1 [%] (*n*)		Dysphagia on Day 7 [%] (*n*)	
	Absent-to-Mild 15–20	Moderate-to-Severe 0–14	Absent-to-Mild 15–20	Moderate-to-Severe 0–14
**Age Groups**				
Left hemisphere stroke				
<75 years	56 (14)	44 (11)	88 (22)	12 (3)
75 and more	33.3 (5)	66.7 (10)	46.7 (7)	53.3 (8)
Right hemisphere stroke				
<75 years	64 (16)	36 (9)	72 (18)	28 (7)
75 and more	53.3 (8)	46.7 (7)	86.7 (13)	13.3 (2)
chi-squared *p*-value	LHS on Day 1	RHS on Day 1	LHS on Day 7	RHS on Day 7
	0.29	0.74	0.01 **	0.49

Statistical significance levels are indicated as follows: ** *p* < 0.01.

**Table 5:** Results of logistic regression analysis for dysphagia severity based on GUSS scores and age threshold (75 years).

Dysphagia Severity	Predictor	Odds Ratio	Lower 95% CI	Upper 95% CI	*p*-Value
Moderate-to-Severe	GUSS 7 Day	0.921	0.829	1.02	0.12
	GUSS (1–7)	1.12	0.941	1.34	0.2
Absent-to-mild	GUSS 7 Day	0.115	0.0107	1.24	0.074
	GUSS (1–7)	0.432	0.199	0.936	0.033 *

Statistical significance levels are indicated as follows: * *p* < 0.05.

**Table 6:** Comparative characteristics of dysphagia, divided by stroke severity according to the NIHSS (National Institutes of Health Stroke Scale).

Variable	Dysphagia on Day 1 [%] (*n*)		Dysphagia on Day 7 [%] (*n*)	
	Absent-to-Mild 15–20	Moderate-to-Severe 0–14	Absent-to-Mild 15–20	Moderate-to-Severe 0–14
NIHSS Scale				
Left hemisphere stroke				
minor stroke	61.5 (8)	38.5 (5)	92.3 (12)	7.7 (1)
moderate stroke	44 (11)	56 (14)	64 (16)	36 (9)
moderate-to-severe stroke	0 ()	100 (2)	50 (1)	50 (1)
Right hemisphere stroke				
minor stroke	85.7 (6)	14.3 (1)	100 (7)	0 ()
moderate stroke	62.5 (15)	37.5 (9)	79.2 (19)	20.8 (5)
moderate-to-severe stroke	40 (2)	60 (3)	60 (3)	40 (2)
severe stroke	25 (1)	75 (3)	50 (2)	50 (2)

**Table 7:** Results of logistic regression analysis for dysphagia and NIHSS severity levels on Day 1 and 7.

Predictor	Odds Ratio	Lower 95% CI	Upper 95% CI	*p*-Value
Day 1				
Intercept	0.203	0	0.816	0.02 *
NIHSS Level	2.11	1	4.17	0.03 *
Day 7				
Intercept	0.0543	0	0.275	0.0004 **
NIHSS Level	2.44	1	5.05	0.016 *

Statistical significance levels are indicated as follows: ** *p* < 0.01, * *p* < 0.05.

**Table 8:** Results of logistic regression analysis for dysphagia and speech disorders.

Predictor	Odds Ratio	Lower 95% CI	Upper 95% CI	*p*-Value
Intercept	0.667	0.354	1.25	0.21
Stroke hemisphere	3.5	1.39	8.84	0.01 **

Statistical significance levels are indicated as follows: ** *p* < 0.01.

**Table 9:** Logistic regression results for patients under 65 years of age: association between thrombolytic treatment and GUSS score improvement.

Predictor	Odds Ratio	Lower 95% CI	Upper 95% CI	*p*-Value
Intercept	6.39	1.35	30.20	0.019 *
Guss (1–7)	1.49	1.01	2.21	0.044 *

Statistical significance levels are indicated as follows: * *p* < 0.05.

**Table 10:** Logistic regression results for specific comorbidities and dysphagia severity.

Comorbidity	GUSS 7 DAY Status (OR)	*p*-Value	GUSS 1–7 Change (OR)	*p*-Value
Previous stroke	1.04	0.43	0.87	0.04 *
Atherosclerosis	0.89	0.04 *	1	0.98
Diabetes	1.01	0.74	1	0.97
Hypertension	1.01	0.90	0.96	0.65
Hypercholesterolemia	1.01	0.85	0.97	0.64
Hypothyroidism	1.1	0.24	1.07	0.46
Post stroke epilepsy	0.93	0.2	1.1	0.51
Degenerative spine disease with discopathy	0.98	0.775	1.1	0.51
Ischemic heart disease	0.89	0.006 **	1.02	0.82
Anemia	1	0.999	0.9	0.20
Atrial fibrillation	0.88	0.004 **	0.96	0.54

Statistical significance levels are indicated as follows: ** *p* < 0.01, * *p* < 0.05.

## Data Availability

The data presented in this study are available on request from the corresponding author.
